# Metabolomics of human fasting: new insights about old questions

**DOI:** 10.1098/rsob.200176

**Published:** 2020-09-16

**Authors:** Hiroshi Kondoh, Takayuki Teruya, Mitsuhiro Yanagida

**Affiliations:** 1 Geriatric unit, Graduate School of Medicine, Kyoto University, Kyoto, Japan; 2 G0 Cell Unit, Okinawa Institute of Science and Technology Graduate University (OIST), Okinawa, Japan

**Keywords:** prolonged fasting, metabolomics, blood metabolites, antioxidant, signalling metabolites, ageing

## Abstract

Since ancient days, human fasting has been performed for religious or political reasons. More recently, fasting has been employed as an effective therapy for weight reduction by obese people, and numerous studies have investigated the physiology of fasting by obese subjects. Well-established fasting markers (butyrates, BCAAs and carnitines) were considered essential energy substitutes after glycogen storage depletion. However, a recently developed metabolomic approach has unravelled previously unappreciated aspects of fasting. Surprisingly, one-third (44) of 120 metabolites investigated increase during 58 h of fasting, including antioxidative metabolites (carnosine, ophthalmic acid, ergothioneine and urates) and metabolites of entire pathways, such as the pentose phosphate pathway. Signalling metabolites (3-hydroxybutyrate and 2-oxoglutarate) and purines/pyrimidines may also serve as transcriptional modulators. Thus, prolonged fasting activates both global catabolism and anabolism, reprogramming metabolic homeostasis.

## 1. History of fasting

Even in ancient Greece, fasting was performed to achieve increased spirituality. Since then, fasting has been adopted as a religious practice by Muslims, Christians, Jews, Buddhists and others [1]. For example, in the tenth century, Sohoh, a buddhist monk in Japan, fasted for 7 days at the end of a thousand-day walk through the mountains from Hieizan to the old Imperial Palace in Kyoto [2]. Subsequently, 50 more people have endured this strict regimen to attain this highest level of priesthood.

Thus, the spiritual and psychological impacts of fasting have been well documented since early times, while health benefits from fasting were noticed only in the nineteenth century. Dr E. H. Dewey, one of the earliest supporters of fasting, claimed erroneously and somewhat hyperbolically that, ‘every disease that afflicts mankind develops from more or less habitual eating in excess of the supply of gastric juices' [3]. In the 1880s, several individual trials of prolonged fasting for 30 to 40 days were reported [4], and we periodically hear news reports of survivors lost at sea or in mountains for weeks. However, most of these are descriptive case reports or anecdotes about physical and metabolic changes in non-obese people.

In 1915, fasting therapy for obesity was described by Folin & Denis [5]. Repeated short periods of fasting were proposed as a safe and effective method of weight reduction [5]. Many obese followers have experienced different regimens of fasting for as much as 100 days or more. Since the 1950s, most data on metabolism during fasting have come from cases involving obesity [6].

Another aspect of human fasting since Roman times concerns hunger strikes. In the UK, suffragettes preformed hunger strikes in the early twentieth century. After the Second World War, Gandhi fasted 14 times or more for up to 21 days as a form of political protest [7]. The longest political fasting of a non-obese person was the case of Terence MacSwiney, a former mayor of Cork, Ireland, who fasted for 74 days to his death, after his arrest during English–Irish unrest in 1920 [8]. Collectively, it appears as though humans can readily survive without any food for 30–40 days, as long as they are properly hydrated.

## 2. Physiology of fasting

**Figure 1.**
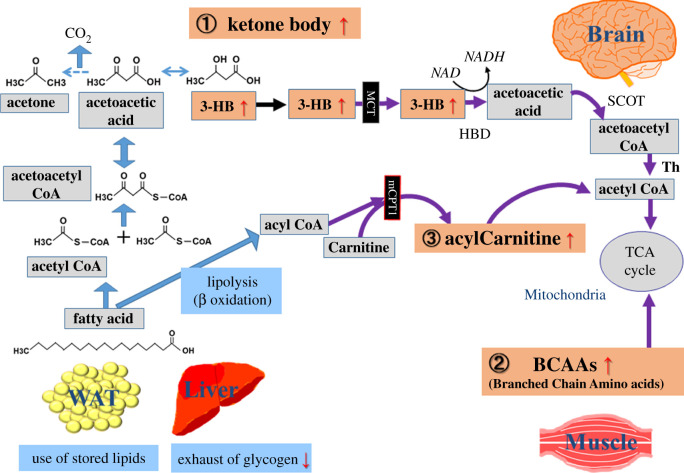
Well-established markers for fasting. After exhaustion of glycogen storage by fasting, lipids in human liver and white adipose tissues (WAT) are used as alternative energy sources. During fasting, 3-hydroxybutyrate (3-HB) is one of the most prominently increased metabolites (over 25-fold), which is generated from acetoacetic acid. Traversing the blood–brain barrier (BBB) via the monocarboxylate transporter (MCT), 3-HB is transported into brain, where fatty acids cannot be used for energy generation. Next, branched chain amino acids (BCAAs) are mainly released from muscles, followed by uptake into the TCA pathway, or lipogenesis in liver. Third, elevated acylcarnitines facilitate lipid transport into mitochondria. Abbreviations: HBD (α-Hydroxybutyric acid dehydrogenase), SCOT (succinyl-CoA:3-oxo-acid CoA transferase), Th (mitochondrial thiolase), mCPT1 (mitochondrial Carnitine palmitoyltransferase I).

Based on the history of fasting, the research on fasting physiology was initiated, focusing on the body's metabolic response, especially in regard to energy substitution. Nutrient limitation, like fasting, significantly affects energy production in the human body, triggering a wide range of catabolic reactions (figure 1). Glucose normally constitutes the major fuel source under non-fasting conditions, but during fasting, glycogen stores are rapidly exhausted in an effort to maintain minimum glucose levels in the blood. After glycogen depletion, constitutive activation of gluconeogenesis supports most glucose production under prolonged fasting [9,10].

In addition to gluconeogenesis, fasting stress forces the human body to use various non-carbohydrate metabolites, such as lipids and branched chain amino acids (BCAAs), as energy sources [11,12]. Hormonal changes, a decrease in plasma insulin concentration and increased catecholamines, stimulate lipolysis in white adipose tissue (WAT) and liver. In lipolysis, 3-hydroxybutyrate (3-HB) increases over 25-fold during fasting. Circulating 3-HB is transported into the brain across the blood–brain barrier (BBB) via the monocarboxylate transporter (MCT). As the brain cannot use fatty acids for energy, unlike most other tissues, 3-HB is converted into acetyl-CoA, providing the brain with an alternative source of energy during prolonged fasting [11]. Succinyl-CoA-3-oxaloacid CoA transferase (SCOT) catalyses the first rate-limiting step in ketolysis by transferring the CoA from succinyl-CoA to acetoacetyl-CoA. SCOT is expressed in all tissues except liver, a major ketogenesis organ, while it is most abundantly expressed in heart, brain and kidney. Elevated acylcarnitines during fasting also are essential for lipid transport into mitochondria [13]. Increased concentrations of BCAAs, mainly released from muscles, are also used in the mitochondrial TCA cycle or in liver lipogenesis [14]. Thus, the elevation of butyrates, BCAAs, and acylcarnitines in circulating blood are well-known indicators of fasting (figure 1).

Lipolytic stimulation facilitates weight reductions of as much as 1–2 kg day^−1^ during fasting, demonstrating that it constitutes an effective therapeutic approach for obese patients. However, prolonged fasting is accompanied by various complications, such as headaches, nausea, weakness, cramps, orthostatic hypotension, and sometimes lethal cardiac arrhythmias, lactic acidosis and renal failure. For fasting research, careful observation and study designs are required.

In addition, the recent advance in ageing research suggests the positive impact of fasting on organismal longevity. In 1934, McCay *et al*. first reported that calorie restriction by 20% expanded lifespans by up to 20% in mice [15]. Calorie restriction of 20–30% can also impact longevity in another experimental model, *Caenorhabditis elegans*, *Drosophila*, fish and monkeys. Calorie restriction modulates several signalling pathways, including sirtuins, AMP kinase and the Tor pathway [16–18]. Genetic and chemical manipulation of these pathways consistently extends organismal lifespan in experimental models. Moreover, intermittent fasting, a cycle of 3 days of fasting and 3 days of feeding, enables *C. elegans* to live about 50% longer than those on a normal diet [19]. Thus, calorie restriction and intermittent fasting are well-established protocols for prolongation of lifespan. However, calorie restriction or fasting studies on longevity of non-obese humans is much more complex. Thus, little is known about the link between fasting and ageing in humans.

## 3. Metabolomics of fasting

Metabolomics, one of the rapidly developing domains of chemical biology, constitutes a powerful tool in the search for useful diagnostic or bio-markers, by quantitative detection of various metabolites. Metabolites, small organic compounds, are generated by the metabolic activity of living organisms from bacteria to humans [20,21]. Today, metabolomic studies provide valuable information about metabolic profiles of tissues, cells, media, fluids and blood. Human blood is especially convenient and useful to analyse, as it circulates throughout the body every few minutes, reflecting *in vivo* physiological states influenced by genetic, epigenetic, physiological and lifestyle factors. Thus, metabolomics of human blood permits comprehensive evaluation of metabolic mechanisms of physiological responses and diseases, and of biological effects of drugs, nutrients and environmental stressors.

Blood comprises cellular and non-cellular components: red blood cells (RBCs), white blood cells (WBCs), platelets and plasma. As fasting is one of the most comprehensive physiological stimuli to the human body, several studies on serum or plasma metabolites during human fasting have been reported. A study by I. Rubio-Aliaga *et al*. monitored 36 h fasting of 10 volunteers with broad range of BMI and age (18.5–39.7 kg m^−2^ and 25–56 years, respectively) [22], while S. Krug *et al*. reported the outcome of 36 h fasting of 15 young, healthy, non-obese participants [23]. These studies consistently identified β-oxidation intermediates, butyrates, BCAAs and acylcarnitines as fasting markers, which are well known as energy substitutes.

We previously established accurate, quantitative procedures to analyse metabolites of human whole blood, plasma and RBCs by liquid chromatography-mass spectrometry (LC-MS), based on our experience in developing metabolomic methods for fission yeast cells under various nutritional and genetic perturbations [24–26]. Our metabolomic approach to whole blood efficiently detects blood metabolites in both in RBCs and plasma. By this approach, we have reported 14 age-related compounds and 15 markers for frailty, a complex disease of cognitive impairment, hypomobility, and decline in normal daily activity, due to age-related dysfunction of tissues and vulnerability to stress [27,28]. These metabolites include large numbers that are enriched in RBCs, confirming the efficacy of whole blood analysis.

**Figure 2.**
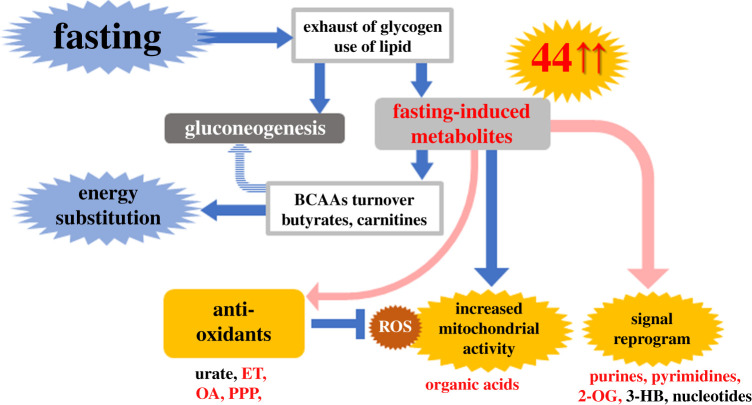
Forty-four metabolites that increase during fasting include antioxidants, organic acids and signalling-related compounds. Non-targeted comprehensive metabolomics of whole blood detected increases of one-third (44) of metabolites identified during 58 h of fasting. In addition to metabolites for energy production, antioxidative metabolites were identified as fasting markers, which may combat oxidative stress resulting from enhanced mitochondrial activity. Moreover, signalling metabolites would contribute for remodelling of metabolic homeostasis during fasting. See the text for details. Abbreviations: ET; ergothioneine, OA; ophthalmic acid, PPP; pentose phosphate pathway, 3-HB; 3-hydroxybutyrate and 2-OG; 2-oxoglutarate.

We performed non-targeted comprehensive LC-MS analysis of whole blood, plasma and RBCs during 58 h fasting by four young, non-obese volunteers, because most metabolic studies of fasting have tracked only specific plasma or serum metabolites, such as butyrates, acylcarnitines and BCAAs [29]. In addition to established fasting markers, several TCA cycle-related compounds (cis-aconitate, malate, 2-oxoglutarate and succinate) and coenzymes (nicotinamide and pantothenate, a precursor for acetyl-CoA) were also increased, reflecting enhanced mitochondrial activity in tissues during fasting. Notably, 44 of 120 metabolites increased 1.5- to 60-fold during this period. Thus, our whole blood metabolomics revealed unexpected dynamics of diverse metabolite increases resulting from greatly activated catabolism and anabolism, stimulated by fasting (figure 2).

## 4. New aspects of fasting: antioxidants and signalling metabolites

It is also conceivable that fasting provokes global remodelling of transcriptional networks to adapt to metabolic changes. Consistently, whole blood metabolomics identified purines and pyrimidines (GTP, CTP, ADP, IMP, cytidine and adenine) and some signal-modulating metabolites (3-HB and 2-oxoglutarate) as fasting markers [29]. The former would support anabolic metabolism for RNA and protein synthesis, while the latter may function as signalling modules to maintain physiological homeostasis during fasting. 3-HB is also known as a histone deacetylase inhibitor, as is the related sodium butyrate [30]. 2-oxoglutarate activates 2-oxoglutarate oxygenase, functioning in demethylation of histones and nucleic acids, and destabilization of transcriptional factors [31]. Fasting may genetically or epigenetically modify transcriptional networks via such metabolites.

In addition to increased metabolites for energy production, previously unappreciated impacts of prolonged fasting were disclosed. Increases of several antioxidants (carnosine, ophthalmic acid (OA), ergothioneine (ET), urate and xanthine) and pentose phosphate pathway (PPP) metabolites were newly observed. These antioxidant metabolites had not been discovered in previous targeted studies on fasting, except for the increase of urate, one of the most abundant antioxidants in blood [32]. Carnosine, OA, ET and urate are known as RBC-enriched compounds, which were efficiently detected by our whole blood metabolomics [27]. Xanthine is the precursor of urate. Carnosine, formed from β-alanine and histidine, is enriched in muscle. OA (L-γ-glutamyl-L-α-aminobutyrylglycine) is a tripeptide analogue of glutathione, in which cysteine is replaced by 2-aminobutyrate (2-AB), another fasting marker. ET is mainly synthesized in mushrooms and other fungi. Among these, increases in antioxidants (OA and ET) during fasting are evolutionarily conserved in both humans and fission yeast [24]. Moreover, the pentose phosphate pathway (PPP) is essential for redox maintenance via NADPH generation. 6-phosphogluconate, glucose-6-phosphate, pentose phosphate and sedoheptulose-7-phosphate are generated in the PPP, levels of which were increased in plasma, but not in RBCs during fasting. Our previous RBC metabolomics identified sugar phosphates compounds as RBC-enriched; therefore, PPP metabolite increases only in plasma suggest that responses in tissues are largely responsible for these altered profiles during fasting.

Collectively, the increased antioxidative defence is a significant physiological response during fasting. Oxidative stress exerts deleterious effects on cells and tissues, while antioxidative defence preserves cellular function and longevity in experimental models. Interestingly, calorie restriction extends organismal lifespans by reducing oxidative damage. Calorie restriction, including fasting, might modulate longevity by boosting antioxidant metabolites and activation of PPP. Alternatively, increased antioxidative metabolism would defend physical homeostasis against the oxidative attack derived from increased mitochondrial activity. In summary, a metabolomic approach to fasting revealed novel aspects of its physiological impacts, which may have clinical applications as diagnostic and therapeutic tools in the future.

## Data accessibility

This article has no additional data.
